# Shiga Toxin–Associated Hemolytic Uremic Syndrome in Adults, France, 2009–2017

**DOI:** 10.3201/eid2707.204638

**Published:** 2021-07

**Authors:** Benoît Travert, Antoine Dossier, Matthieu Jamme, Aurélie Cointe, Yahsou Delmas, Sandrine Malot, Alain Wynckel, Amélie Seguin, Claire Presne, Miguel Hie, Ygal Benhamou, David Ribes, Gabriel Choukroun, Steven Grangé, Alexandre Hertig, Emilie Cornec Le Gall, Lionel Galicier, Eric Daugas, Lila Bouadma, François-Xavier Weill, Elie Azoulay, Fadi Fakhouri, Agnès Veyradier, Stéphane Bonacorsi, Julien Hogan, Véronique Frémeaux-Bacchi, Eric Rondeau, Patricia Mariani-Kurkdjian, Paul Coppo, Centre de Référence des Microangiopathies Thrombotiques

**Affiliations:** Centre de Référence des Microangiopathies Thrombotiques, Paris, France (B. Travert, A. Dossier, M. Jamme, Y. Delmas, S. Malot, A. Wynckel, A. Seguin, C. Presne, M. Hie, Y. Benhamou, G. Choukroun, S. Grangé, A. Hertig, L. Galicier, E. Azoulay, F. Fakhouri, A. Veyradier, V. Frémeaux-Bacchi, E. Rondeau, P. Coppo);; Université de Paris, Paris (B. Travert, A. Dossier, A. Cointe, L. Galicier, E. Daugas, L. Bouadma, E. Azoulay, A. Veyradier, S. Bonacorsi, J. Hogan, V. Frémeaux-Bacchi, P. Mariani-Kurkdjian);; Hôpital Bichat—Claude Bernard, Paris (B. Travert, A. Dossier, E. Daugas, L. Bouadma);; Sorbonne-Université, Paris (M. Jamme, M. Hie, A. Hertig, E. Rondeau, P. Coppo);; Hôpital Tenon, Paris (M. Jamme, E. Rondeau);; Hôpital Robert-Debré, Paris (A. Cointe, S. Bonacorsi, J. Hogan, P. Mariani-Kurkdjian);; Centre Hospitalier Universitaire de Bordeaux, Bordeaux, France (Y. Delmas);; Hôpital Maison Blanche, Reims, France (A. Wynckel);; Centre Hospitalier Universitaire de Caen, Caen, France (A. Seguin);; Centre Hospitalier Universitaire d’Amiens, Amiens, France (C. Presne, G. Choukroun);; Groupement Hospitalier Pitié-Salpêtrière, Paris (M. Hie, A. Hertig);; Centre Hospitalier Universitaire de Rouen, Rouen, France (Y. Benhamou, S. Grangé);; Centre Hospitalier Universitaire de Toulouse, Toulouse, France (D. Ribes);; Centre Hospitalier Universitaire de Brest, Brest, France (E. Cornec-Le Gall);; Hôpital Saint-Louis, Paris (L. Galicier, E. Azoulay); I; nstitut Pasteur, Paris (F.-X. Weill);; Centre Hospitalier Universitaire de Nantes, Nantes, France (F. Fakhouri);; Hôpital Lariboisière, Paris (A. Veyradier);; Hôpital Européen Georges Pompidou, Paris (V. Frémeaux-Bacchi);; Hôpital Saint Antoine, Paris (P. Coppo)

**Keywords:** hemolytic uremic syndrome, Shiga toxin, thrombotic microangiopathy, Escherichia coli, E. coli, bacteria, Shiga toxin–producing Escherichia coli, STEC, enteric infections, food-borne infections, food safety, HUS, France

## Abstract

We conducted a retrospective study on hemolytic uremic syndrome caused by Shiga toxin–producing *Escherichia coli* (STEC) in 96 adults enrolled in the cohort of the National Reference Center for Thrombotic Microangiopathies network in France during 2009–2017. Most infections were caused by STEC strains not belonging to the O157 or O104 serogroups. Thirty (31.3%) patients had multiple risk factors for thrombotic microangiopathy. In total, 61 (63.5%) patients required dialysis, 50 (52.1%) had a serious neurologic complication, 34 (35.4%) required mechanical ventilation, and 19 (19.8%) died during hospitalization. We used multivariate analysis to determine that the greatest risk factors for death were underlying immunodeficiency (hazard ratio 3.54) and severe neurologic events (hazard ratio 3.40). According to multivariate analysis and propensity score-matching, eculizumab treatment was not associated with survival. We found that underlying conditions, especially immunodeficiency, are strongly associated with decreased survival in adults who have hemolytic uremic syndrome caused by STEC.

Shiga toxin–producing *Escherichia coli* (STEC) infection is an environmental foodborne or waterborne disease that causes bloody diarrhea. Approximately 5%–20% of cases are complicated by hemolytic uremic syndrome (HUS) (*1*,*2*). Shiga toxins (Stx) can cause acute microvascular injury, leading to thrombotic microangiopathy (TMA), which is characterized by hemolytic anemia and thrombocytopenia, and in the scenario of HUS, associated with acute kidney injury (*3*). Researchers estimate that the global prevalence of STEC infection is ≈43.1 acute illnesses/100,000 person-years, causing ≈3,890 annual cases of STEC-associated HUS (*4*). STEC-associated HUS occurs mostly in children; sporadic cases are rare in adults.

Among children, STEC-associated HUS is the most frequent form of TMA and the leading cause of acute renal failure (*3*). In France, surveillance for STEC-associated HUS in children <15 years of age has existed since 1996. This surveillance system comprises 32 pediatric healthcare centers, including all 21 university hospital units specializing in pediatric nephrology. These centers notify public health authorities of cases of STEC-associated HUS. The National Reference Center for *Escherichia coli*, *Shigella* and *Salmonella* at the Institut Pasteur (NRC-Ec; Paris, France) and its associated laboratory at the Robert Debré University Hospital (Paris, France) confirm and characterize STEC infections in children and adults. This surveillance network estimated the annual incidence of HUS in France to be 1.00 case/100,000 child-years, causing a ≈1% death rate during 2007–2016 (*5*).

Despite the much lower incidence of HUS among adults than children, most deaths caused by STEC-associated HUS occur among persons >60 years of age (*2*,*6*). The French national health authorities do not have a dedicated surveillance system for STEC-associated HUS in adults. In 2011, a large STEC outbreak in Europe sickened 3,816 persons in Germany, causing 845 cases of HUS and 54 deaths; 24 persons were affected in the Bordeaux region of France, including 9 who had HUS, 8 of whom were adults (*7*,*8*). The outbreak was linked to an atypical hybrid pathotype *E. coli* O104:H4 strain characterized by enteroaggregative and enterohemorrhagic virulence; the strain also produced an extended spectrum β-lactamase. Most (88%) patients involved in this outbreak, which was associated with consumption of organic fenugreek sprouts, were adults, and the median age was 42 years. Publicity surrounding this outbreak raised awareness of STEC-associated HUS in adults. However, cases of STEC-associated HUS in adults remain rare (*9*,*10*). Hence, the clinical characteristics of adult STEC-associated HUS and the effects of therapeutic strategies on outcome remain uncertain. We describe the epidemiologic and clinical features of adults with STEC-associated HUS, identify predictors of patient outcomes, and assess the effectiveness of therapeutic interventions in this population.

## Methods

### Study Design, Settings, and Data Sources

We conducted a retrospective cohort study of STEC-associated HUS cases in adults registered during January 2009–December 2017 in France by the Centre National de Référence des Microangiopathies Thrombotiques (CNR-MAT; https://www.cnr-mat.fr). We reviewed all medical files from the CNR-MAT database. This work was part of the TMA study approved by our institutional review board (Comité pour la protection des personnes Ile-de-France; approval no. CPP04807) in accordance with the Declaration of Helsinki and the French Data Protection Authority.

### Diagnostic Criteria

The diagnosis of HUS required the coexistence of TMA (i.e., thrombocytopenia [platelet levels <150,000 cells/μL] and microangiopathic hemolytic anemia [hemoglobin levels <12 g/dL]) and an acute kidney injury (AKI). We included all TMA patients >18 years of age in the CNR-MAT cohort who had an AKI and a positive PCR result for the Stx genes *stx1*, *stx2*, or both*.* We considered patients to have fever if they had a temperature of >38°C within 24 hours after admission.

### Microbiological Data

Participating laboratories conducted PCR specific for *stx1* and *stx2* on *E. coli* strains isolated from stool, blood, and urine samples. Laboratory technicians also cultured samples from *stx*-positive stools. To characterize the isolated STEC strains, technicians used an O-serogroup multiplex PCR selective for the 10 most frequent serogroups affecting humans in France: O157, O26, O145, O55, O103, O104, O111, O91, O121, and O80 (*11*). Strains belonging to other serogroups were characterized by PCR of the restriction fragment length polymorphism of the O operon, *rfb* (*rfb-*RFLP) (*12*). In April 2017, NRC-Ec and local laboratories also began to characterize strains using whole-genome sequencing, when available. If a strain was *stx*-positive but its serogroup was not identified by culture, we classified that strain as not isolated.

### Variables

Participating laboratories and physicians submitted data on each patient’s medical history, clinical and biological features, microbiological findings, and treatment at admission and during hospitalization (*13*). We retrospectively calculated each patient’s age-weighted Charlson Comorbidity Index (CCI) (*14*) and classified AKI according to the Kidney Disease: Improving Global Outcomes (KDIGO) criteria published by the International Society of Nephrology (*15*). We investigated ADAMTS13 and complement alternative pathway (CAP) activity as previously described (*16*).

### Treatments and Outcomes

Treatment consisted mainly of therapeutic plasma exchange (TPE) or best supportive care (BSC) according to the discretion of the treating physician. The C5 complement blocker eculizumab (Soliris; Alexion Pharmaceuticals, Inc., https://alexion.com) also was given at the discretion of the treating physician; however, physicians were encouraged to discuss eculizumab use with a member of the CNR-MAT team. The primary outcome of this study was patient survival at the time of most recent follow-up.

### Statistics

We reported qualitative variables as frequencies and percentages; we reported quantitative discrete and continuous variables as medians and interquartile ranges (IQRs). We estimated survival using the Kaplan-Meier method. We used Cox proportional hazards regression to identify factors independently associated with survival. The proportional hazard assumption was supported by a nonsignificant relationship between scaled Schoenfeld residuals and time and refuted by a significant relationship using an alpha (α) risk set at 5%. We reported the results using hazard ratios (HRs) and 95% CIs, using an α risk set at 5% statistical significance. To quantify the effect of eculizumab on survival, we calculated and compared the propensity scores of patients who did and did not use eculizumab (Appendix). We used R software version 3.6.1 (The R Project for Statistical Computing, https://www.r-project.org) for statistical analysis. For propensity score analysis, we used MatchIt package (*17*).

## Results

**Figure 1:**
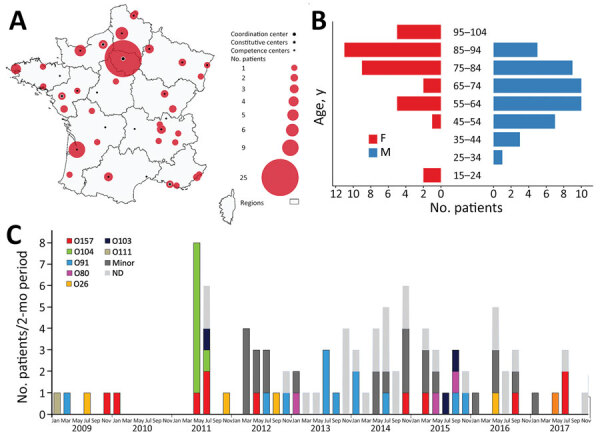
Distribution of adults with Shiga toxin–associated hemolytic uremic syndrome, France, 2009–2017. A) Geographic distribution of cases and thrombotic microangiopathy reference centers. The Centre National de Référence des Microangiopathies Thrombotiques is a national network comprising 1 coordination center, 5 constitutive centers, and 21 competence centers. B) Age and sex distribution of cases. C) Bimonthly distribution of cases according to serogroup. Of patients with minor serogroups, 4 had strains belonging to O106, 3 to O128, 3 to O174, 2 to O113, 1 to O100, 1 to O126, 1 to O148, 1 to O177, 1 to O78, 1 to O84, and 7 to an O serogroup not typable at the time of identification. ND, not determined.

Of the 4,048 patients in the CNR-MAT cohort, we first identified 61 adult STEC-associated HUS patients with complete data during January 2009–December 2017. After comparing the NRC-Ec and CNR-MAT surveillance data, we identified 35 additional patients to be included in the study cohort. In total, the study cohort comprised 96 patients (Appendix Figure 1). This cohort included patients from hospitals throughout France, most of which were part of the CNR-MAT network (Figure 1, panel A). The women-to-men ratio was 1.7 and median age was 60.5 years (IQR 47.0–71.0 years) (Figure 1, panel B). Geographic, temporal, and microbiological characteristics of the cases suggested an outbreak among 13 patients (Figure 1). The cohort also included 8 patients affected by the 2011 O104:H4 outbreak in France described previously (*8*). We found a patient in our cohort who was infected in a family cluster of STEC-associated HUS in 2014, but the strain could not be identified. We also found 4 patients (2 in Marne, 1 in Nord, 1 in Paris) who tested positive for STEC O91 in summer 2013 but did not share a known infection source.

In total, 69 (71.9%) patients had underlying conditions; the median CCI was 2.00 (IQR 1.00–4.25) (Table 1). Of the 96 patients, 27 (28.1%) had an underlying immunodeficiency and 30 (31.3%) had >1 condition that might contribute to TMA.

**Table 1:** Characteristics of adults with Shiga toxin–associated hemolytic uremic syndrome, France, 2009–2017*

Characteristic	Value
Median age, y (IQR)	60.5 (47.00–71.00)
Sex	
M	35 (36.5)
F	61 (63.5)
Median age-weighted Charlson Comorbidity Index (IQR)	2.00 (1.00–4.25)
Tobacco use within previous 3 y	12 (12.5)
>1 underlying condition	69 (71.9)
Cardiovascular disease	48 (50.0)
Arterial hypertension	38 (39.6)
Diabetes mellitus	12 (12.5)
Venous thromboembolic disease	11 (11.5)
Heart disease†	20 (20.8)
CKD‡	15 (15.6)
History of kidney transplant	5 (5.2)
Stage 2 CKD	4 (4.2)
Stage 3 CKD	8 (8.3)
Stage 4 CKD	3 (3.1)
Digestive disorder§	29 (30.2)
Gastrointestinal disorder	18 (18.8)
Biliopancreatic disorder	9 (9.4)
Hepatic disorder	4 (4.2)
Autoimmune or inflammatory disease¶	11 (11.5)
Immunodeficiency	27 (28.1)
History of bone marrow or solid organ transplant#	8 (8.3)
Hematologic disease**	8 (8.3)
Active cancer††	8 (8.3)
HIV‡‡	3 (3.1)
Primary immunodeficiency§§	2 (2.1)
Neuropsychiatric disorder¶¶	18 (18.8)
Treatment	
Immunosuppressive treatment	12 (12.5)
Corticosteroids	11 (11.5)
Calcineurin inhibitors	7 (7.3)
Azathioprine or mycophenolate mofetil	7 (7.3)

*Values are no. (%) patients except as indicated. CKD, chronic kidney disease; IQR, interquartile range. †8 patients had hypertensive disease, 4 had ischemic disease, 4 had hypertensive and ischemic disease, 2 had valvular cardiopathy, 1 had pulmonary hypertension, and 1 had unspecified heart disease. ‡According to Kidney Disease Improving Global Outcomes guidelines (*15*). §8 patients had gastric, small bowel, or colonic resection; 2 had history of bariatric surgery; 3 had chronic diarrhea from diverticulosis; 1 had graft-versus-host disease; 1 had colonic endometriosis; 1 had microscopic colitis; 1 had AA amyloidosis; 1 had neurovegetative disorder (1 each); 3 had recurrent pyogenic cholangitis; 1 had sclerosing cholangitis; 2 had a double kidney-pancreas transplantation; 3 had chronic pancreatitis; 2 had cirrhosis; 1 had history of liver transplant; and 1 had autoimmune hepatitis. ¶2 patients had mixed connective tissue disease, 1 had systemic sclerosis, 1 had sclerosing cholangitis, 1 had microscopic polyangiitis, 3 had type 1 diabetes, 1 had multiple sclerosis, and 2 had psoriasis. #3 patients had a history of kidney, 2 of double kidney–pancreas, 2 of bone marrow, and 1 of liver transplant. **1 patient had acute myeloid leukemia, 1 had chronic lymphocytic leukemia, 1 had Hodgkin’s lymphoma, 1 had clonal B-cell lymphocytosis, 1 had monoclonal gammopathy of undetermined significance, 1 had Waldenström’s disease, 1 had myeloproliferative disorder, and 1 had myelodysplastic syndrome. ††3 patients had breast cancer, 1 had metastatic lung cancer, 1 had a gastrointestinal stromal cell tumor, 1 had bladder cancer, 1 had cervical cancer, and 1 had gastric cancer. ‡‡3 patients had AIDS, including 2 patients who received HIV diagnoses during treatment. §§2 patients had hypogammaglobulinemia, including 1 patient who had ICF1 syndrome caused by a DNMT3b germinal mutation. ¶¶5 patients had stroke sequelae, 3 had Parkinson’s disease, 1 had multiple sclerosis, 4 had cognitive impairment (including 1 patient who had Korsakoff syndrome and 1 who had vascular dementia), 1 had epilepsy, 1 had chronic polyradiculoneuropathy, and 5 had major depressive or bipolar disorder.

Most (83.3%) patients had diarrhea and nearly half (49.0%) had bloody diarrhea; 11 patients had severe colitis, including 4 who required emergency surgery (Table 2). All patients had renal impairment. In 2011, 2 patients with STEC O104:H4 infection had proteinuria (i.e., >1 g/L) but not serum creatinine elevation; these patients also had microangiopathic hemolytic anemia and peripheral thrombocytopenia (*8*). The other 94 patients all had AKI stage 1 or higher according to KDIGO criteria, of which 61 (63.5%) required dialysis. Of 12 patients who underwent kidney biopsy, 11 showed signs of TMA. Most (76%) patients had neurologic symptoms, mainly confusion (56.3%) and headache (18.8%). Approximately half (52.1%) of patients had a serious neurologic complication such as seizure, coma, or focal deficiency. In addition, 34 (35.4%) patients required mechanical ventilation. In total, 42 patients had high blood pressure (>150/90 mm Hg) at admission; severe hypertension (>170/110 mm Hg) subsequently developed in 11 patients and hypertensive retinopathy developed in 6 patients. Only 2 patients had hypotension (<90/60 mm Hg) at admission. In total, 41 (42.7%) patients had cardiac events; in 26 of 43 cases with available data, patients had troponin levels above the defined threshold of their respective laboratory (Table 2).

**Table 2:** Clinical and biological characteristics of adults with Shiga toxin–associated hemolytic uremic syndrome, France, 2009–2017*

Characteristic	Value
Clinical features	
Fever	16/78 (20.5)
Diarrhea	80/96 (83.3)
Bloody diarrhea	47/96 (49.0)
Median time between symptom onset and hospitalization, d (IQR)†	4 (1–9)
Renal manifestations	
Median serum creatinine level, μmol/L (IQR)‡	221.5 (145–391)
Acute kidney injury§	96/96 (100.0)
KDIGO stage 1	3/96 (3.1)
KDIGO stage 2	17/96 (17.7)
KDIGO stage 3	74/96 (77.1)
Hemodialysis	61/96 (63.5)
Median time before dialysis, d¶	3 (1–6)
Oligoanuria	47/96 (49.0)
Proteinuria (i.e., >0.5 g/L)	33/42 (78.6)
Neurologic events	
Any neurologic sign	73/96 (76.0)
Headache	18/96 (18.8)
Confusion	54/96 (56.3)
Seizure	30/96 (31.3)
Coma	36/96 (37.5)
Focal deficiency	25/96 (26.0)
Abnormal brain imaging#	23/58 (39.7)
Abnormal computed tomographic scan	5/38 (13.2)
Abnormal magnetic resonance imaging	20/42 (47.6)
Stroke, coma, or seizure	50/96 (52.1)
Required mechanical ventilation	34/96 (35.4)
Cardiac events	
Any cardiac event	41/96 (42.7)
High troponin level	26/43 (60.5)
Acute coronary syndrome	4/96 (4.2)
New onset of heart arrhythmia	4/96 (4.2)
Acute heart failure	16/96 (16.7)
Recovered from cardiac arrest	1/96 (1.0)
Laboratory features	
Median leukocyte count, 10^9^ cells/L (IQR)	10.5 (8.05–13.8)
Median platelet count, 10^9^ cells/L (IQR)**	56 (36–120)
Platelet count <30, 10^9^ cells/L**	19/96 (19.8)
Platelet count <30, 10^9^ cells/L and serum creatinine <200 μmol/L**	5/96 (5.2)
Median hemoglobin, g/dL (IQR)**	10.4 (8.90–12.4)
Elevated lactate deshydrogenase (i.e., >250 IU/L)	78/79 (98.7)
C-reactive protein, mg/L (IQR)††	48 (22–120)
Elevated lipase (i.e., >210 U/L)	5/32 (15.6)
Low CH50 (i.e., <70%)	26/67 (38.8)
Low C3 (i.e., <0.660 g/L)	5/69 (7.2)
Low C4 (i.e., <0.093 g/L)	3/69 (4.3)
Low complement factor H (i.e., <70%)	3/69 (4.3)
Low complement factor I (i.e., <70%)	1/69 (1.4)
Low CD46 (i.e., <13 µg/L)	23/35 (65.7)
Anti-FH antibody (>150 arbitrary units)‡‡	2/69 (2.9)
Microbiological findings	
Shiga toxin genotype	96/96 (100)
*stx1*+/*stx2*–	12/84 (14.3)
*stx2*+/*stx1*–	63/84 (75.0)
*stx1+/stx*2*+*	9/84 (10.7)
*Escherichia coli* serogroup	
O157	10/67 (14.9)
O104	8/67 (11.9)
O91	12/67 (17.9)
O80	4/67 (6.0)
O26	4/67 (6.0)
O103	3/67 (4.5)
O111	1/67 (1.5)
Minor serogroups§§	25/67 (37.3)
Isolation site	
Stool	90/96 (93.8)
Urine	7/96 (7.3)
Blood culture	4/96 (4.2)
Multisite¶¶	5/96 (5.2)

*Values are no. with characteristic/total no. patients (%) except as indicated. KDIGO, Kidney Disease Improving Global Outcomes (*15*). †Time of symptom onset was the first reported day of diarrhea (for patients with diarrhea) or fever, confusion, abdominal pain, or nausea/vomiting (for patients without diarrhea). ‡Out of 90 patients; samples taken at admission. §Two patients had proteinuria (i.e., >1 g/L) but not elevation of serum creatinine. ¶Out of 60 patients requiring dialysis. #Recent brain lesions on magnetic resonance imaging, computed tomographic scan, or both. Fourteen patients had stroke lesions, 3 had posterior reversible encephalopathy syndrome lesions, 4 had intracerebral bleeds, and 12 had white matter lesions consistent with thrombotic microangiopathies. **Samples taken at admission. ††Out of 52 patients. ‡‡One patient had 242 arbitrary units and 1 had 800 arbitrary units. §§Four patients had strains belonging to O106, 3 to O128, 3 to O174, 2 to O113, 1 to O100, 1 to O126, 1 to O148, 1 to O177, 1 to O78, 1 to O84, and 7 to an O serogroup not typable at the time of identification. ¶¶Two patients had *E. coli* in urine and stool samples, 2 had *E. coli* in stool and blood samples, and 1 had *E. coli* in blood and urine samples.

CAP measurements during the acute phase of illness were recorded in 69 patients. Of these patients, 36 (52.2%) had values within the reference range (Table 2). Less than 10% of patients had low levels of C3, C4, factor H, or factor I, whereas 26 (38.8%) patients had low levels of CH50. CD46 levels were low in 65.7% (23/35) patients. Two patients had low levels of anti-factor H antibodies (242 and 800 arbitrary units) (Table 2). ADAMTS13 activity was detectable (>10%) in all 69 patients in whom it was tested. 

Among the 84 cases in which *stx* type was detected, *stx1*–/*stx2*+ was the most common genotype (85.7%). The *stx1*+/*stx2*– genotype was significantly associated with increased CCI and immunodeficiency (Appendix Table 1). As expected, the most common STEC isolation site was stool (93.8%), whereas only 10 patients had STEC-positive urine or blood samples. Seven (7.3%) patients had STEC-positive urine samples, including 5 who had a urologic infection without associated colitis. Four patients had STEC-positive blood samples, including 1 patient for whom STEC was identified in blood samples only. In total, 5 patients had a multisite infection. 

Most (60; 62.5%) had a serogroup typable by the NRC-Ec; 7 (7.3%) patients had an untypable serogroup. The most common serogroups were O91 (12; 17.9%) and O157 (10; 14.9%) (Table 2). The STEC isolates from urine samples belonged to the O104, O91, O106, O126, O174, and O148 serogroups; isolates from blood samples belonged to the O80, O103, and O128 serogroups (Appendix Table 2).

**Figure 2:**
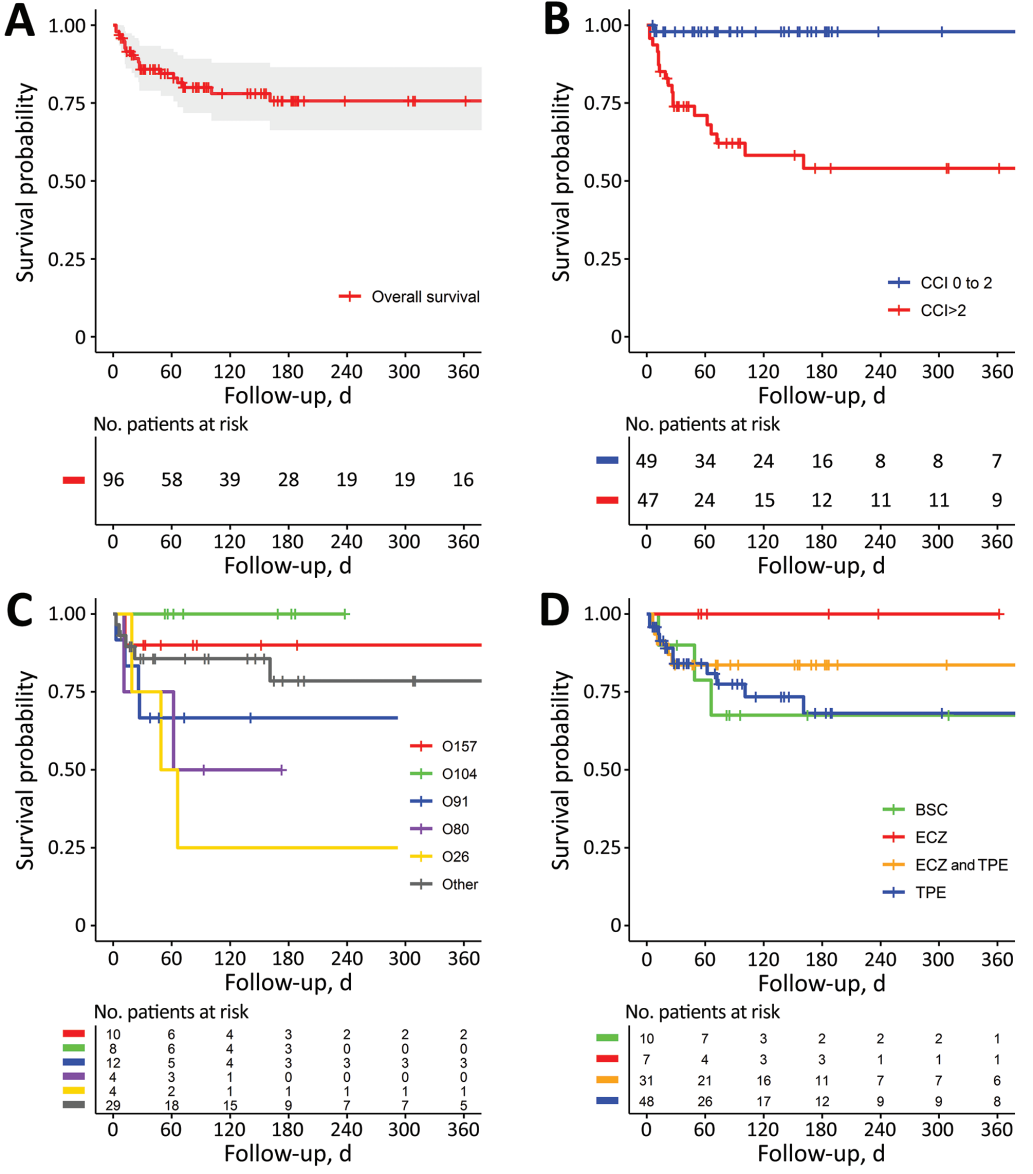
Kaplan-Meier survival plots of adults with Shiga toxin–associated hemolytic uremic syndrome, France, 2009–2017. A) Overall. B) By age-weighted Charlson comorbidity index. C) By STEC serogroup. D) By treatment. Plots show time from admission to death. p values determined using log-rank test. BSC, best standard of care; CCI, age-weighted Charlson comorbidity index; ECZ, eculizumab; TPE, therapeutic plasma exchange.

In total, 19 (19.8%) patients died during hospitalization (Figure 2, panel A; Appendix Table 3). Patients died 3–152 days after admission and had a median follow-up period of 112 days (IQR 49–238). After follow-up, 1 patient had HELLP (hemolysis, elevated liver enzymes, low platelets) syndrome; the patient was STEC-negative at the time of the episode. None of the surviving patients had a further episode of TMA during follow-up.

Patients were treated mainly with BSC, TPE, or eculizumab; 3 patients also received immunoadsorption treatment (Appendix Table 4). Of the 61 patients who required dialysis, 17 (25.4%) died. At the end of the follow-up period, 6 (9.8%) patients still required dialysis, including 4 who had a follow-up period of >90 days. Patients who received dialysis were treated for a median duration of 13.5 days (IQR 8–28 days); 38 patients no longer required dialysis at the end of the follow-up period. After a median follow-up period of 34 days (IQR 23–75 days), the median serum creatinine value was 92 μmol/L (IQR 74–124 μmol/L). Of the 50 patients with a severe neurologic complication, 14 (28.0%) died. Of the 25 surviving patients with available data, 8 (32.0%) patients had neurologic sequelae, including persistent sensorimotor deficit (7, 28.0%), epilepsy (2, 8.0%), and cognitive impairment (2, 8.0%).

In total, 26 (27.1%) patients were treated with macrolides, including 3 who received the treatment to prevent infectious meningoencephalitis associated with eculizumab. Fifty-seven (59.4%) patients received β-lactam antimicrobial drugs, aminoglycosides, or quinolones; 22 (22.9%) patients received metronidazole.

After unadjusted analysis, we found that age (HR 1.04, 95% CI 1.01–1.07; p = 0.01), CCI (HR 1.15, 95% CI 1.03–1.28; p = 0.02) (Figure 2, panel B), underlying immunodeficiency (HR 4.36, 95% CI 1.72–11.07; p<0.01), and associated digestive disease (HR 4.07, 95% CI 1.63–10.14; p<0.01) were significantly associated with death of all causes (Table 3). We also found that severe neurologic events (HR 2.90, 95% CI 1.04–8.06; p = 0.04), mechanical ventilation (HR 2.71, 95% CI 1.09–6.74; p = 0.03), and dialysis (HR 5.57, 95% CI 1.29–24.16; p = 0.02) were predictive of death. High troponin levels and *stx* types were not associated with survival (Table 3). Most patients who died had STEC strains belonging to non-O104 and non-O157 serogroups (Figure 2, panel C; Appendix Table 3). We found that overall survival was comparable among patients treated by different combinations of BSC, TPE, and eculizumab (p = 0.43 by log-rank test) (Table 3; Figure 2, panel D). The use of macrolides was not associated with survival (p = 0.77).

**Table 3:** Survival analysis of 96 adults with Shiga toxin–associated hemolytic uremic syndrome, France, 2009–2017*

Characteristic	Outcome			Univariate analysis			Multivariate analysis†	
	Survived	Died		Unadjusted HR (95% CI)	p value		Adjusted HR (95% CI)	p value
Demographic data								
Median age, y (IQR)‡	58.0 (43.0–68.0)	69.0 (58.5–78.0)		1.04 (1.01–1.07)	0.01		1.03 (1.00–1.06)	0.09
Age quartile, y								
20–47	24 (31.2)	1 (5.3)		1	Ref			
47–60.5	18 (23.4)	5 (26.3)		6.31 (0.74–54.05)	0.09			
60.5–71	21 (27.3)	4 (21.1)		4.64 (0.52–41.50)	0.17			
71–99	14 (18.2)	9 (47.4)		12.93 (1.63–102.31)	0.02			
Sex								
M	26 (33.8)	9 (47.4)						
F	51 (66.2)	10 (52.6)		0.66 (0.27–1.63)	0.37		0.36 (0.13–1.02)	0.06
Medical history								
>1 underlying condition	50 (64.9)	19 (100.0)			<0.01§			
Median age-weighted CCI (IQR)‡	2.0 (0.0–4.0)	5.0 (3.0–6.0)		1.15 (1.03–1.28)	0.02			
Digestive disease	18 (23.4)	11 (57.9)		4.07 (1.63–10.14)	<0.01		2.04 (0.70–5.90)	0.19
Cardiovascular disease	36 (46.8)	12 (63.2)		2.04 (0.80–5.19)	0.14		1.31 (0.43–3.98)	0.63
Heart disease	14 (18.2)	6 (31.6)		1.75 (0.66–4.59)	0.26			
Renal disease	12 (15.6)	3 (15.8)		0.87 (0.25–3.01)	0.83			
Neurologic disease	12 (15.6)	6 (31.6)		1.94 (0.74–5.11)	0.18		1.14 (0.37–3.52)	0.81
Autoimmune disease	8 (10.4)	3 (15.8)		1.45 (0.42–4.97)	0.56			
Immunodeficiency	15 (19.5)	12 (63.2)		4.36 (1.72–11.07)	<0.01		3.54 (1.24–10.14)	0.02
Indicators of organ involvement								
Median platelet count, × 10^9^ cells/L (IQR)‡¶	56.0 (32.5–122.0)	59.5 (45.8–95.0)		1.00 (1.00–1.00)	0.83			
Median serum creatinine, µmol/L (IQR)¶	222 (140–396)	221 (190–352)		1.00 (1.00–1.00)	0.45			
Stage 3 acute kidney injury#	55 (71.4)	19 (100.0)			0.01§			
Dialysis	44 (57.1)	17 (89.5)		5.57 (1.29–24.16)	0.02		3.49 (0.77–15.79)	0.10
Stroke, coma, or seizure	36 (46.8)	14 (73.7)		2.90 (1.04–8.06)	0.04		3.40 (1.05–11.04)	0.04
Mechanical ventilation	23 (29.9)	11 (57.9)		2.71 (1.09–6.74)	0.03			
High troponin**	19 (54.3)	7 (87.5)		5.06 (0.62–41.14)	0.13			
Microbiological findings								
Shiga toxin genotypes††								
*stx1*+/*stx2*–	8 (12.1)	4 (22.2)		1	Ref			
*stx2*+/*stx1*–	50 (75.8)	13 (72.2)		0.64 (0.21–1.96)	0.43			
*stx1+/stx*2*+*	8 (12.1)	1 (5.6)		0.31 (0.04–2.81)	0.30			
Serogroup‡‡					0.05§			
O157	9 (17.3)	1 (6.7)						
O104	8 (15.4)	0						
O91	8 (15.4)	4 (26.7)						
O26	1 (1.9)	3 (20.0)						
O80	2 (3.8)	2 (13.3)						
Other	24 (46.2)	5 (33.3)						
Treatments								
BSC	7 (9.1)	3 (15.8)		1.52 (0.44–5.23)	0.51			
TPE	63 (81.8)	16 (84.2)		1.25 (0.36–4.29)	0.72			
ECZ	33 (42.9)	5 (26.3)		0.49 (0.18–1.36)	0.17			
Time from admission to ECZ treatment, d§§								
<7	16 (50.0)	2 (66.7)						
>7	16 (50.0)	1 (33.3)						
Macrolides	21 (27.3)	5 (26.3)		0.86 (0.31–2.39)	0.77			
Other antimicrobial drugs¶¶	43 (55.8)	14 (73.7)		2.02 (0.73–5.62)	0.18			
Therapeutic strategy##					0.43§			
BSC	7 (9.1)	3 (15.8)						
TPE without ECZ	37 (48.1)	11 (57.9)						
ECZ without TPE	7 (9.1)	0						
TPE and ECZ	26 (33.8)	5 (26.3)						

*Values are no. (%), except as indicated. BSC, best supportive care; CCI, age-weighted Charlson Comorbidity Index; ECZ, eculizumab; HR, hazard ratio; IQR, interquartile range; ref, referent; TPE, therapeutic plasma exchange. †Akaike Information Criterion = 149.20; 19 events. ‡Shapiro-Wilk Normality test was used to check normality for age distribution (p = 0.30), CCI (p<0.01), and platelet count (in logarithmic form; p = 0.41 by log-normal distribution). §By log-rank test. ¶Samples taken at admission. #According to Kidney Disease Improving Global Outcomes criteria (*15*). **According to upper thresholds defined by respective laboratories. Out of 43 patients with known troponin level. ††Out of 84 patients with known Shiga toxin subgroups, 18 died. ‡‡Out of 67 patients with known serogroups, 15 died. §§The first day of ECZ administration was not reported in the medical records of 3 patients. ¶¶Including β-lactams, quinolones, and aminoglycosides. ##Multivariate comparison of BSC, TPE without ECZ, ECZ without TPE, and ECZ+TPE treatment regimens.

Multivariate analysis showed that underlying immunodeficiency (HR 3.54, 95% CI 1.24–10.14; p = 0.02) and severe neurologic events (HR 3.40, 95% CI 1.05–11.04; p = 0.04) were negatively associated with survival (Table 3). After adjustment of determinants retained for the multivariate analysis, we found that eculizumab was not associated with survival (HR 0.77, 95% 0.25–2.33; p = 0.64). Propensity score-matching also indicated that eculizumab was not associated with survival (p = 0.34) (Appendix Table 5, Figure 2).

## Discussion

We found that 20% of adults who had STEC-associated HUS died during hospitalization, in agreement with previous findings (*9*,*10*); however, <1% of children who had STEC-associated HUS died in France during the same years, 2007–2016 (*5*). In addition, adults had cerebral involvement 3 times more frequently than children (*2*); 52.1% of adult patients had severe neurologic manifestations, similar to the observations of Karpac et al. (*10*). Renal recovery was slow and inconsistent; 4 patients still required dialysis 90 days after hospitalization (*9*). One third of patients required mechanical ventilation. These findings emphasize that, in adults, STEC-associated HUS is a severe systemic disease that can cause multiple organ failure. However, inclusion in the CNR-MAT registry relied on voluntary physician reporting; thus, this case series is not exhaustive and might disproportionately reflect the most severe cases. As previously observed for children (*18*), most cases in this cohort were sporadic and, for unclear reasons, in women. In regard to age distribution, STEC-associated HUS has a U curve from birth to old age (*6*,*9*,*10*,*19*). During the study period, 1,095 STEC-associated HUS cases in children were reported to Santé Publique France through the country’s pediatric surveillance network (*5*). By comparison, this disease appears to be much rarer among adults, although underreporting is probable.

Our findings on underlying conditions and deaths by age group resemble those of the FoodNet registry of elderly adults with STEC-associated HUS (*9*). The risk for death from STEC-associated HUS increases for persons age >40 years, suggesting that young and middle-aged adults have similar clinical courses to those observed in children. We found a strong association between underlying conditions and decreased survival, especially for patients with immunodeficiency (*9*,*20*–*23*). The prevalence of antibodies against Stx decreases for persons >40 years of age (*24*), which might account for the more severe forms of STEC-associated HUS in elderly persons. The expression of glomerular globotriaosylceramide (Gb3), the main receptor of Stx, was thought to decrease with age; however, researchers now believe that expression levels remain stable throughout a person’s lifetime (*25*). Renal and neurologic signs similar to those caused by HUS develop in immunocompromised mice after STEC inoculation or Stx exposure, whereas wild-type mice are naturally resistant to this disease (*26*,*27*). Together, these findings highlight the role of the immune system in preventing STEC-associated HUS. Immunodeficiency probably contributes to disease severity.

The 2011 outbreak in Europe illustrated that microbiological characteristics play a key role in STEC-associated HUS (*7*). The distribution of serotypes among adults in our study was slightly different than in a study on pediatric HUS in France in the same timeframe (*5*). Non-O157 strains were more prevalent in the pediatric series (*5*) and in ours, whereas O157 and O26 were more commonly observed among children than adults (23% among children vs. 15% among adults for O157; 11% among children vs. 6% among adults for O26) (*5*). A similar overall distribution was observed among children and adults with STEC infection in Norway (23% for O157, 10% for O26) (*28*). By contrast, serogroups O91 and O104 have been mainly found among adults (*29*,*30*). The data might have been skewed by the 2011 outbreak caused by a strain belonging to the O104 serogroup; this outbreak caused infections in younger persons who had fewer underlying conditions, which could account for the better outcomes of those patients. Other serogroups, especially O80, O26, and O91, are emerging and might be associated with increased pathogenicity (*2*,*18*). STEC O91 was also the most common serogroup among adults with STEC infections in Germany (*30*), which raises the question of increased pathogenicity in adults and in persons >40 years of age.

In agreement with previous reports of STEC-associated HUS in adults (*29*,*31*), we found that *stx1+/stx2*– strains were more prevalent among adults (14.3%) than had been previously documented among children (2.0%) (*5*). One possible explanation for this distribution might be that in some patients, HUS was concurrent with but unrelated to infection or colonization by *stx1+/stx2*– STEC; however, this scenario is unlikely because STEC-positive patients had typical features of HUS in an infectious context. We cannot exclude the possibility that the *stx2* gene could have been lost in human hosts during infection or ex vivo during subculture, as already described for STEC O26 (*32*). In this series, all *stx1+/stx2*– strains belonged to non-O157 serogroups. These findings are similar to those of Käppeli et al. (*29*), who found that 15.8% of cases of non-O157 STEC–associated HUS were caused by *stx1+/stx2*– strains, which could suggest that different serogroups might pose different risks for HUS associated with particular *stx* genotypes. Last, most (83%) patients with *stx1+/stx2*– genotypes had underlying immunodeficiency; one explanation could be that immunodeficient patients are more susceptible to Stx1. The alleles *stx1* and *stx2c* have been associated with a lower risk for severe STEC infection and HUS (*28*). However, *stx1a* is associated with higher risk for severe STEC infection (*33*). We did not have data on *stx* subtypes in our study.

We observed CAP abnormalities similar to those previously reported in a cohort of 113 cases of STEC-associated HUS in children (*16*). We found that 65.7% of patients had low CD46 and 38.8% had low CH50 levels. However, a decrease in the concentration of complement factors, the interpretation of which remains equivocal, might be attributable to kidney damage and STEC-associated HUS (*16*). The presence of an inflammatory syndrome further complicates the interpretation of these data. In contrast to atypical HUS, pediatric STEC-associated HUS has not been linked to a constitutional or acquired dysregulation of the CAP. Screening for variants in complement genes is not usually conducted among children with STEC-associated HUS. Similarly, it seems unlikely that STEC infection reveals underlying CAP abnormalities in many adults.

We found that 7% of patients had STEC-positive urine samples, an underrecognized finding documented by Lavrek et al. (*34*). Although urine samples might be easily contaminated, especially in patients who have diarrhea, these findings encourage systematic STEC-specific PCR screening and culture confirmation of stool or other biological samples (in the event of extraintestinal *E. coli* infection) from adult TMA patients (*2*,*34*).

Because the effectiveness of specific treatments remains unclear, BSC is the cornerstone of STEC-associated HUS treatment (*2*,*35*,*36*). Univariate analysis indicated that TPE was not associated with overall survival improvement, although other studies have concluded differently (*37*–*39*). However, considering the substantial overlap between the signs and symptoms of STEC-associated HUS in adults and TMA of other etiologies, some researchers believe that plasma therapy should be given until TTP or atypical HUS are ruled out (*13*,*40*). Whether TPE should be continued after the determination of *stx* status remains unclear. As previously reported, we did not find a clear survival benefit from eculizumab (*38*,*41*). However, the small sample size and the strong differences between patients who did and did not receive eculizumab treatment preclude definitive conclusions.

The benefits of antimicrobial drugs in treating STEC-associated HUS are unclear (*42*,*43*). Previous studies suggest that the use of antimicrobial drugs during early stages of STEC infection is associated with the development of HUS. However, the effects of antimicrobial drugs administered after HUS diagnosis remain unknown (*42*). A retrospective study reported that azithromycin administered during STEC infection might reduce the duration of STEC carriage (*43*). We found that use of macrolides was not associated with survival. This observation might have been confounded by possible unreported administration of antimicrobial drugs before hospitalization, treatment for unstandardized indications at the discretion of the practitioner, or other variables. We also found that the prescription of multiple antimicrobial drugs was a common practice, especially in cases of severe infection.

In conclusion, STEC-associated HUS is rarer among adults than among children but causes more severe disease and death. Underlying conditions, especially immunodeficiency, are strongly associated with decreased survival. The severity of the disease, a probably underestimated prevalence, and the risk for outbreaks of emerging STEC-associated HUS provide strong arguments for active epidemiologic and microbiological surveillance of this disease.

Appendix Additional information on Shiga toxin–associated hemolytic uremic syndrome in adults, France, 2009–2017.
