# Supplementary material for: Shiga Toxin–Associated Hemolytic Uremic Syndrome in Adults, France, 2009–2017
Source: Emerg Infect Dis. 2021 Jul;27(7):1876–85. doi: 10.3201/eid2707.204638 (PMC8237898; doi:10.3201/eid2707.204638)
Supplement: Appendix — Additional information on Shiga toxin–associated hemolytic uremic syndrome in adults, France, 2009–2017. [file 20-4638-Techapp-s1.pdf]

Article DOI: https://doi.org/10.3201/eid2707.204638

Shiga Toxin–Associated Hemolytic and Uremic Syndrome in Adults, France, 2009–2017

Appendix

Appendix Methods

We used logistic regression to estimate the distance measure for propensity score-matching. We used the nearest neighbor method with caliper index equal to 0.3 to conduct 1:1 matching, because this method balanced the number of unmatched patients and acceptable comparability between matched groups. We created a random computerized loop to optimize best matching for our final analysis. Other methods, including exact, stratification matching, or many-to-one matching on the propensity score, did not enable the formation of acceptable comparison groups because patients treated with eculizumab had different characteristics that appeared to be the main determinants of survival (Appendix Table 6; Appendix Figure 2).

Appendix Table 1. Clinical and biological characteristics of 96 adults with Shiga toxin–associated hemolytic uremic syndrome by infecting strain genotype, France, 2009–2017*

	stx genotype			
Characteristic	*stx1*+/*stx2*–†	*stx1*–/*stx2*+‡	*stx1*+/*stx2*+§	p value¶
Total	12	63	9	
Clinical features				
Sex				
M	5 (41.7)	24 (38.1)	3 (33.3)	
F	7 (58.3)	39 (61.9)	6 (66.7)	1.00
Median age, y (IQR)	52.50 (42.75–60.25)	62.00 (51.00–71.00)	56.00 (43.00–64.00)	0.24
Median age-weighted	6.00 (4.75–7.25)	2.00 (0.50–4.00)	2.00 (0.00–3.00)	<0.01
Charlson comorbidity index (IQR)				
≥1 Underlying condition	12 (100.0)	46 (73.0)	6 (66.7)	0.07
Immunodeficiency	10 (83.3)	15 (23.8)	1 (11.1)	<0.01
Digestive disorder	8 (66.7)	15 (23.8)	3 (33.3)	0.02
Cardiovascular disease	10 (83.3)	29 (46.0)	4 (44.4)	0.06
Diarrhea	9 (75.0)	54 (85.7)	6 (66.7)	0.12
Bloody diarrhea	5 (41.7)	29 (46.0)	5 (55.6)	0.87
Isolation site				
Stool	10 (83.3)	61 (96.8)	8 (88.9)	0.10
Urine	1 (8.3)	4 (6.3)	1 (11.1)	0.63
Blood	2 (16.7)	1 (1.6)	0	0.08
Events and outcomes				
Dialysis	9 (75.0)	40 (63.5)	5 (55.6)	0.60
Stroke, coma, or seizure	6 (50.0)	33 (52.4)	5 (55.6)	1.00
Any cardiac event	6 (50.0)	23 (36.5)	6 (66.7)	0.22
Death	4 (33.3)	13 (20.6)	1 (11.1)	0.45

*Values are no. (%), except as indicated.
†Three *stx1*-positive strains belonged to serogroup O103, 3 to O26, 1 to O111, 1 to O126, 1 to O128, 1 to O78, and 1 to O84; 1 strain was not typable.
‡Twelve *stx2*-positive strains belonged to serogroup O91, 9 to O157, 8 to O104, 4 to O106, 4 to O80, 2 to O113, 2 to O128, 2 to O174, 1 to O100, 1 to O148, 1 to O177, and 1 to O26; 3 strains were not typable. Remaining strains were not isolated or not available.
§One *stx1*-positive/*stx2*-positive strain belonged to serogroup O157 and 1 belonged to O174; 3 strains were not typable.
¶P values were determined by Fisher exact test for categorical variables and Kruskal-Wallis test for continuous variables. Remaining strains were not isolated or not available.

Appendix Table 2. Clinical and biological characteristics of 96 adults with Shiga toxin–associated hemolytic uremic syndrome by serogroup, France, 2009–2017*

	Serogroup											
Characteristic	O91	O157	O104	O26	O80	O106	O103	O128	O174	Other	Not isolated†	Not available‡
Total	12	10	8	4	4	4	3	3	3	16	17	12
Clinical features												
Sex												
M	5 (41.7)	4 (40.0)	2 (25.0)	3 (75.0)	4 (100.0)	2 (50.0)	1 (33.3)	1 (33.3)	0	5 (31.3)	5 (29.4)	3 (25.0)
F	7 (58.3)	6 (60.0)	6 (75.0)	1 (25.0)	0	2 (50.0)	2 (66.7)	2 (66.7)	3 (100.0)	11 (68.8)	12 (70.6)	9 (75.0)
Median age, y (IQR)	70.0 (67.5–74.75)	63.5 (47.0–71.25)	41.0 (36.25–47.5)	48.0 (37.25–59.25)	59.0 (52.5–62.75)	69.5 (61.75–76.75)	56.0 (54.0–56.5)	61.0 (46.5–80.0)	44.0 (38.0–65.5)	55.5 (46.0–62.5)	66.0 (54.0–73.0)	62.5 (55.75–72.5)
Median age-weighted Charlson comorbidity index (IQR)	3.0 (2.0–4.25)	3.5 (0.25–4.0)	0.0	6.0 (5.5–6.5)	3.5 (2.25–4.5)	2.5 (1.75–3.75)	4.0 (2.5–5.0)	2.0 (1.0–5.5)	0.0 (0.0–4.0)	3.0 (1.0–5.0)	2.0 (1.0–5.0)	2.0 (1.0–3.25)
≥1 Underlying condition	12 (100.0)	6 (60.0)	1 (12.5)	4 (100.0)	4 (100.0)	3 (75.0)	3 (100.0)	2 (66.7)	2 (66.7)	13 (81.3)	14 (82.4)	5 (41.7)
Immunodeficiency	3 (25.0)	1 (10.0)	0	4 (100.0)	2 (50.0)	0	2 (66.7)	1 (33.3)	0	6 (37.5)	7 (41.2)	1 (8.3)
Digestive disorder	3 (25.0)	0	0	1 (25.0)	2 (50.0)	1 (25.0)	2 (66.7)	1 (33.3)	1 (33.3)	7 (43.8)	8 (47.1)	3 (25.0)
Cardiovascular disease	8 (66.7)	4 (40.0)	0	2 (50.0)	2 (50.0)	3 (75.0)	3 (100.0)	0	1 (33.3)	12 (75.0)	8 (47.1)	5 (41.7)
Diarrhea	8 (66.7)	10 (100.0)	8 (100.0)	4 (100.0)	4 (100.0)	3 (75.0)	3 (100.0)	2 (66.7)	2 (66.7)	9 (56.3)	16 (94.1)	11 (91.7)
Bloody diarrhea	4 (33.3)	8 (80.0)	5 (62.5)	2 (50.0)	3 (75.0)	0	2 (66.7)	1 (33.3)	1 (33.3)	4 (25.0)	9 (52.9)	8 (66.7)
Isolation site												
Stool	12 (100.0)	10 (100.0)	8 (100.0)	4 (100.0)	4 (100.0)	3 (75.0)	3 (100.0)	2 (66.7)	2 (66.7)	14 (87.5)	17 (100.0)	11 (91.7)
Urine	1 (8.3)	0	1 (12.5)	0	0	1 (25.0)	0	0	1 (33.3)	2 (12.5)	0	1 (8.3)
Blood	0	0	0	0	1 (25.0)	0	1 (33.3)	1 (33.3)	0	0 (0.0)	0	1 (8.3)
stx genotypes												
stx1+/*stx2*–	0	0	0	3 (75.0)	0	0	3 (100.0)	1 (33.3)	0	5 (31.3)	0	NA
stx2+/*stx1*–	12 (100.0)	9 (90.0)	8 (100.0)	1 (25.0)	4 (100.0)	4 (100.0)	0	2 (66.7)	2 (66.7)	8 (50.0)	13 (76.5)	NA
stx1+/*stx2*+	0	1 (10.0)	0	0	0	0	0	0	1 (33.3)	3 (18.8)	4 (23.5)	NA
Events and outcomes												
Dialysis	11 (91.7)	6 (60.0)	2 (25.0)	3 (75.0)	3 (75.0)	3 (75.0)	3 (100.0)	2 (66.7)	2 (66.7)	7 (43.8)	12 (70.6)	7 (58.3)
Stroke, coma, or seizure	8 (66.7)	5 (50.0)	3 (37.5)	1 (25.0)	3 (75.0)	3 (75.0)	2 (66.7)	2 (66.7)	1 (33.3)	10 (62.5)	6 (35.3)	6 (50.0)
Any cardiac event	4 (33.3)	3 (30.0)	3 (37.5)	2 (50.0)	2 (50.0)	2 (50.0)	1 (33.3)	0	1 (33.3)	9 (56.3)	8 (47.1)	6 (50.0)
Death	4 (33.3)	1 (10.0)	0	3 (75.0)	2 (50.0)	1 (25.0)	1 (33.3)	0	0	3 (18.8)	3 (17.6)	1 (8.3)

*Values are no. (%), except as indicated. Major serogroups (i.e., O91, O157, O26, O80, O103, O111) comprised 62.1% of identified strains.
†Strain not identified.
‡Identification not performed.

Appendix Table 3. Characteristics of 19 adults who died of Shiga toxin–associated hemolytic uremic syndrome, France, 2009–2017*

Pt	Age, y	Sex	Underlying conditions	Serogroup	*stx* type	Minimum platelet count, 10^9 cells/L	Packed red blood cells, nb†	Dialysis	Mechanical ventilation	Severe neurologic complications	Cardiac event	Tr	Days to death	Cause(s) of death
1	73	M	Colonic surgery, short bowel syndrome, hypogammaglobulinemia	O91	*stx*2	75	0	+	+	Coma, stroke	AHF	TPE	3	MOF
2	87	F	COPD, pulmonary hypertension, AH	O91	*stx*2	20	2	+				TPE	12	Sepsis
3	69	M	Mixed connective tissue disease	O91	*stx*2	19	4	+	+	Seizure, soma, stroke	Elevated troponin	TPE, ECZ	26	SNS
4	80	M	AH, COPD, ICM	O91	*stx*2	8	NA	+	+	Seizure, coma		TPE	27	Sepsis, VAP
5	73	F	Parkinson’s disease with dementia	O157	*stx*2	15	4	+		Seizure, oma	Elevated troponin	BSC	12	SNS
6	57	M	Bone marrow transplantation (for refractory anemia with excess of blasts), digestive graft-versus-host disease	O80	*stx*2	3	+	+	+	Coma	HArr, AHF	TPE, ECZ	11	MOF, gastrointestinal bleeding
7	68	M	Bone marrow transplant (for acute myeloid leukemia), digestive graft-versus-host disease	O80	*stx*2	NA	0					TPE	62	Progressive graft-versus-host disease
8	78	F	Kidney transplantation (for ANCA-associated vasculitis), AH, ICM, renal insufficiency, depression, basocellular carcinoma	O26	*stx*1	63	2	+				TPE	19	MOF, sepsis, and gastrointestinal bleeding
9	53	M	Waldenström macroglobulinemia, COPD, cachexia	O26	*stx*2	77	0		+	Seizure, coma		BSC	66	SNS
10	20	M	Congenital immunodeficiency (immunodeficiency, centromeric region instability, facial anomalies syndrome; DNMT3b mutation), chronic colitis, autoimmune hepatitis, AH, renal insufficiency	O26	*stx*1	61	6	+			Elevated troponin, AHF	BSC	49	MOF
11	57	F	Liver transplant (for hepatitis B and C), CAFIB, AH, COPD, depression	O103	*stx*1	39	17	+		Stroke	HArr	TPE	152	SNS, persistent renal failure
12	79	F	Gastric cancer, stroke	O106	*stx*2	NA	0	+		Seizure, coma, stroke		TPE	3	SNS
13	65	F	AH, DM, ICM, CAFIB, primary sclerosing cholangitis	O177	*stx*2	29	+	+	+	Seizure, coma	Elevated troponin, AHF	TPE, ECZ	6	MOF
14	60	F	Cervical cancer, DM	Onew H27	*stx*2	32	3	+	+	Seizure, coma	AHF	TPE	44	MOF
15	68	M	Colon surgery, AH	NA	*stx*	42	2	+	+	Seizure, coma, stroke	Elevated troponin	TPE, ECZ	8	SNS
16	53	M	Chronic lymphocytic leukemia, chronic biliary disease, renal insufficiency, AH	NA	*stx*1	13	+	+	+	Seizure, coma, stroke	Elevated troponin	TPE, ECZ	22	SNS
17	80	F	Breast and endometrial cancer, AH, DM, ICM, CAFIB	NA	*stx*2	16	5	+			HArr, AHF	TPE	27	AHF caused by TPE
18	78	F	Parkinson’s disease with severe dysautonomia	NA	*stx*1+2	32	9	+	+	Seizure, coma, stroke	Elevated troponin	TPE	72	SNS and VAP
19	73	F	Lung cancer, depression	NA	*stx*2	67	11	+	+	coma		TPE	101	Sepsis, VAP, cancer

*AH, arterial hypertension; AHF, acute heart failure; BSC, best standard of care; CAFIB, chronic atrial fibrillation; COPD, chronic obstructive pulmonary disease; DM, diabetes mellitus; ECZ, eculizumab; HArr, heart arrythmia; ICM, ischemic cardiomyopathy; MOF, multiple organ failure; NA, not available; Pt, patient; SNS, severe neurologic sequelae; TPE, therapeutic plasma exchange; Tr, treatment; VAP, ventilation-associated pneumonia.
†Values reported where available.

Appendix Table 4. Clinical characteristics and outcomes of adults with Shiga toxin–associated hemolytic uremic syndrome by treatment, France, 2009–2017*

Characteristic	Best supportive care	TPE only	ECZ only	ECZ and TPE	ECZ, TPE, and immunoadsorption
Total	10 (100.0)	48 (100.0)	7 (100.0)	28 (100.0)	3 (100.0)
Medical history					
Median age, y (IQR)	64.5 (48.5–75.25)	62.0 (55.75–73.0)	38.0 (35.5–44.0)	57.5 (48.75–68.0)	64.0 (59.0–64.50)
Sex					
M	4 (40.0)	20 (41.7)	2 (28.6)	9 (32.1)	0
F	6 (60.0)	28 (58.3)	5 (71.4)	19 (67.9)	3 (100.0)
Median age-weighted Charlson Comorbidity Index (IQR)	3.5 (2.25–5.75)	3.0 (1.0–5.25)	0.0 (0.0–0.0)	2.0 (0.0–3.25)	2.0 (1.5–2.50)
≥1 Underlying condition	10 (100.0)	38 (79.2)	2 (28.6)	18 (64.3)	1 (33.3)
Digestive disorder	3 (30.0)	20 (41.7)	0	6 (21.4)	0
Cardiovascular disease	8 (80.0)	27 (56.3)	1 (14.3)	12 (42.9)	0
Renal disease	5 (50.0)	7 (14.6)	0	3 (10.7)	0
Immunodeficiency	4 (40.0)	15 (31.3)	1 (14.3)	7 (25.0)	0
Autoimmune or inflammatory disease	4 (40.0)	17 (35.4)	1 (14.3)	8 (28.6)	1 (33.3)
Transplant	0	6 (12.5)	0	2 (7.1)	0
Neuropsychiatric disorder	3 (30.0)	9 (18.8)	2 (28.6)	4 (14.3)	0
Clinical features					
Diarrhea	7 (70.0)	40 (83.3)	7 (100.0)	23 (82.1)	3 (100.0)
Bloody diarrhea	3 (30.0)	24 (50.0)	4 (57.1)	14 (50.0)	2 (66.7)
Laboratory features					
Median platelet count, 10^9 cells/L (IQR)†	77.5 (53.75–98.75)	51.0 (27.0–95.0)	241.0 (197.5–255.5)	55.0 (33.0–89.0)	36.0 (30.0–162.50)
Median minimum platelet count, 10^9 cells/L (IQR)†	55.0 (35.0–73.0)	32.0 (18.0–50.0)	84.0 (46.0–86.5)	29.5 (21.75–46.75)	29.0 (22.0–33.0)
Median hemoglobin, g/dL (IQR)†	11.55 (9.67–12.25)	10.0 (9.05–11.7)	14.70 (12.65–16.08)	11.0 (8.5–12.4)	12.1 (10.35–13.15)
Renal manifestations					
Median serum creatinine, μmol/L (IQR)†	320.5 (142.0–385.0)	200.0 (157.0–328.0)	83.0 (66.75–217.75)	283.5 (160.5–452.75)	195.0 (130.0–218.00)
Stage 3 acute kidney injury‡	8 (80.0)	34 (70.8)	3 (42.9)	27 (96.4)	2 (66.7)
Required dialysis	4 (40.0)	30 (62.5)	2 (28.6)	24 (85.7)	1 (33.3)
Neurologic events					
Any neurologic events	4 (40.0)	39 (81.3)	3 (42.9)	24 (85.7)	3 (100.0)
Stroke, coma, or convulsions	4 (40.0)	23 (47.9)	2 (28.6)	18 (64.3)	3 (100.0)
Convulsions	2 (20.0)	12 (25.0)	1 (14.3)	12 (42.9)	3 (100.0)
Coma	2 (20.0)	16 (33.3)	0	15 (53.6)	3 (100.0)
Focal neurologic deficit	0	11 (22.9)	2 (28.6)	11 (39.3)	1 (33.3)
Abnormal brain imaging§	2 (66.7)	12 (41.4)	0	8 (40.0)	1 (33.3)
Required mechanical ventilation	1 (10.0)	16 (33.3)	0	14 (50.0)	3 (100.0)
Cardiac events					
Any cardiac event	7 (70.0)	18 (37.5)	1 (14.3)	12 (42.9)	3 (100.0)
High troponin¶	4 (100.0)	11 (57.9)	1 (33.3)	9 (60.0)	1 (50.0)
Serotype O104:H4	0	0	5 (71.4)	2 (7.1)	1 (33.3)
Isolation site					
Stool	9 (90.0)	45 (93.8)	7 (100.0)	26 (92.9)	3 (100.0)
Urine	1 (10.0)	3 (6.3)	1 (14.3)	2 (7.1)	0
Blood	0	3 (6.3)	0	1 (3.6)	0
>1 site	0	3 (6.3)	1 (14.3)	1 (3.6)	0
Other treatments					
Macrolides	1 (10.0)	9 (18.8)	5 (71.4)	9 (32.1)	2 (66.7)
Corticoids	2 (20.0)	11 (22.9)	0	2 (7.1)	1 (33.3)
Outcomes					
Deceased	3 (30.0)	11 (22.9)	0	5 (17.9)	0
Median duration of hospitalization, d (IQR)	19.0 (11.0–24.5)	30.0 (13.0–48.0)	16.0 (11.0–21.0)	38.0 (28.0–47.0)	40.0 (37.5–61.0)
Duration of dialysis >90 d#	1 (50.0)	2 (11.1)	0	1 (5.3)	0
Neurologic sequelae at the end of follow-up **	0	4 (36.4)	0	2 (18.2)	2 (66.7)

Characteristic	Best supportive care	TPE only	ECZ only	ECZ and TPE	ECZ, TPE, and immunoadsorption

*Values are no. (%), except as indicated. ECZ, eculizumab; TPE, therapeutic plasma exchange.
†Samples taken at admission.
‡According to Kidney Disease Improving Global Outcomes criteria (*15*).
§Of 58 patients who had brain imaging, 3 were treated with the best standard of care, 29 with TPE only, 3 with ECZ only, 20 with ECZ and TPE, and 3 with ECZ, TPE, and immunoadsorption.
¶Of 53 patients who had known blood troponin levels, 4 were treated with the best standard of care, 19 with TPE only, 3 with ECZ only, 15 with ECZ and TPE, and 2 with ECZ, TPE, and immunoadsorption.
#Of 42 surviving dialysis patients with available data on the duration of dialysis, 2 were treated with the best standard of care, 18 with TPE only, 2 with ECZ only, 19 with ECZ and TPE, and 1 with ECZ, TPE, and immunoadsorption.
**Of 25 surviving patients with available data who had stroke, coma or convulsions, 11 were treated with TPE only, 11 with ECZ and TPE, and 3 with ECZ, TPE, and immunoadsorption.

Appendix Table 5. Comparison of Shiga toxin–associated hemolytic uremic syndrome patients treated with and without eculizumab, France, 2009–2017*

Characteristic	Eculizumab		p value†
	No	Yes	
Total	58	38	
Matched	30	30	
Unmatched	28	8	
Propensity score variables			
Median age, y (IQR)	59.50 (48.75–71.00)	54.50 (41.00–64.75)	0.19
Median age-weighted Charlson comorbidity index (IQR)	2.00 (0.00–3.75)	1.00 (0.00–3.75)	0.41
Immunodeficiency	8 (26.7)	8 (26.7)	1.00
Dialysis	21 (70.0)	19 (63.3)	0.79
Stroke, coma, or convulsions	17 (56.7)	16 (53.3)	1.00
Therapeutic plasma exchange	27 (90.0)	23 (76.7)	0.30
Other variables			
Death attributable to Shiga toxin–associated hemolytic uremic syndrome	7 (23.3)	4 (13.3)	0.51
Median follow-up time, d (IQR)	99.50 (31.75–181.25)	156.00 (56.00–227.50)	0.38

*Values are no. (%), except as indicated. Values are out of matched scores. Comparison subgroups were made after matching with propensity score (nearest neighbor method; caliper index equal to 0.3).
†p values for categorical variables determined by Fisher exact test; p values for continuous variables determined by Kruskal-Wallis test.

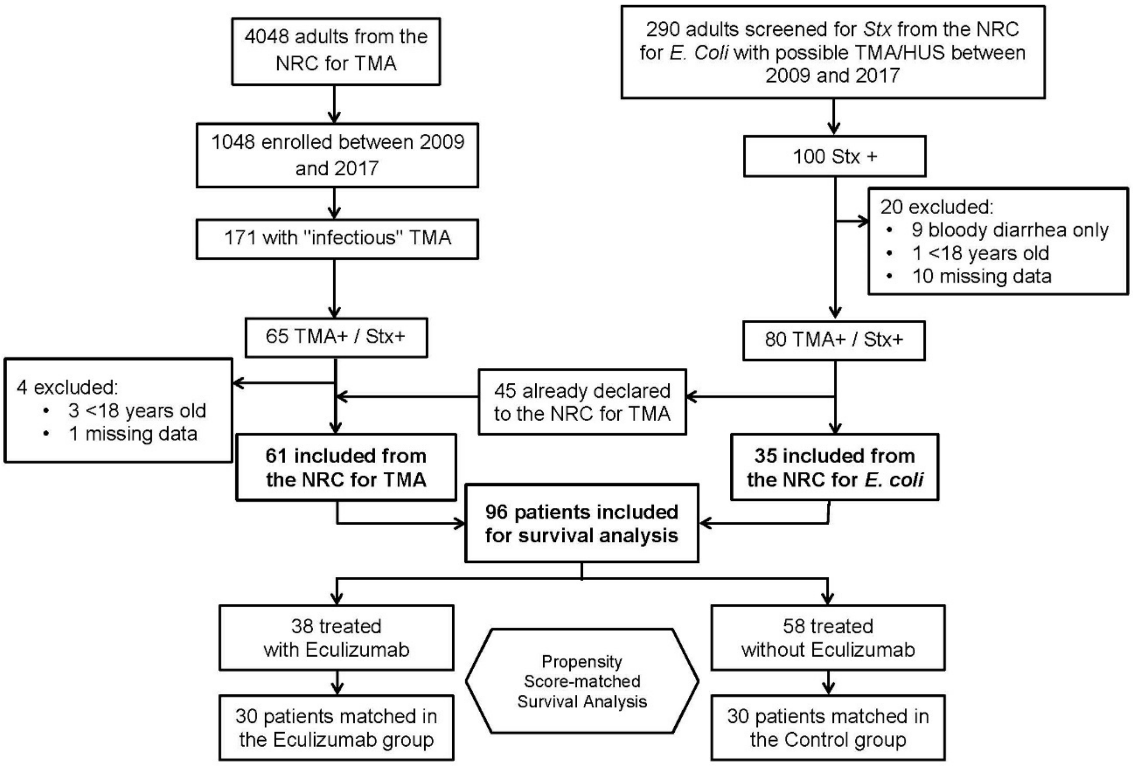

Appendix Figure 1. Design of study on adults with Shiga toxin–associated hemolytic uremic syndrome, France, 2009–2017. NRC, National Reference Centre; *E. coli*, *Escherichia coli*; HUS, hemolytic uremic syndrome; TMA, thrombotic microangiopathy.

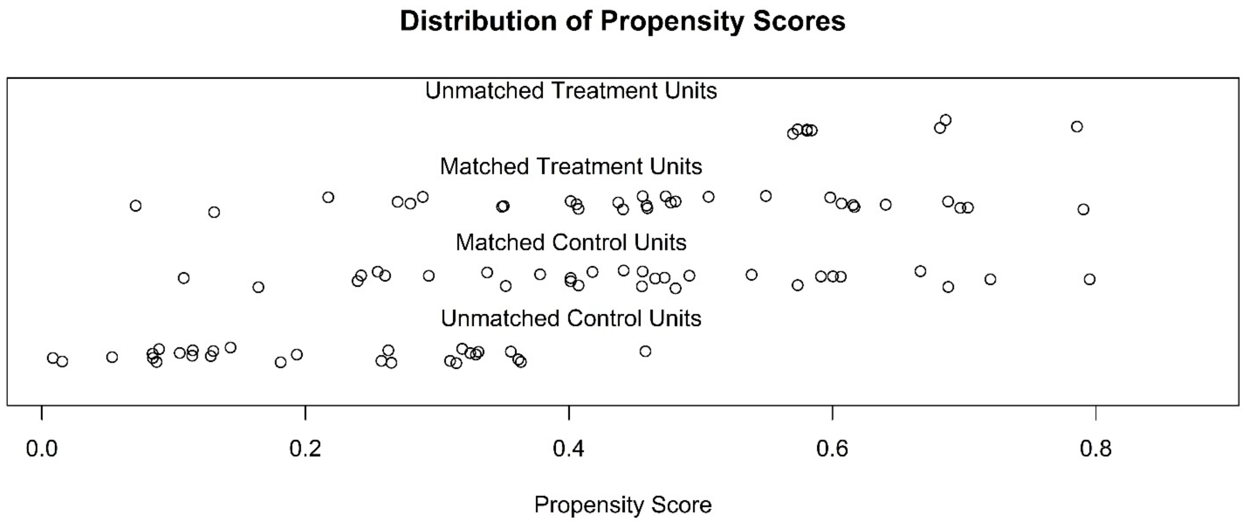

Appendix Figure 2. Distribution of propensity scores of adults with Shiga toxin–associated hemolytic uremic syndrome, France, 2009–2017.
